# Dissecting the biological complexity of age-related macular degeneration: Is it one disease, multiple separate diseases, or a spectrum?

**DOI:** 10.1016/j.exer.2025.110304

**Published:** 2025-02-19

**Authors:** Jason M.L. Miller, Benjamin R. Thompson, James T. Handa, Philip Luthert, Usha Chakravarthy, Karl G. Csaky, Alan Bird, Benjamin K. Young, Sudha K. Iyengar, Jiwon Baek, Moussa A. Zouache, Burt T. Richards, Gregory S. Hageman, Gerry Rodrigues, Kapil Bharti, John G. Flannery, Michael B. Gorin, Catherine Bowes Rickman

**Affiliations:** a University of Michigan, Kellogg Eye Center and Cellular and Molecular Biology Program, Ann Arbor, MI, USA; b Northwestern University Feinberg School of Medicine, Department of Ophthalmology and Feinberg Cardiovascular and Renal Research Inst., Chicago, IL, USA; c Johns Hopkins Medical School, Wilmer Eye Institute, Baltimore, MD, USA; d UCL Institute of Ophthalmology, London, United Kingdom; e Queens University of Belfast, Center for Public Health, Belfast, Ireland; f Retina Foundation of the Southwest, Dallas, TX, USA; g Oregon Health Sciences University, Department of Ophthalmology, Portland, OR, USA; h Case Western Reserve University, Department of Population and Quantitative Health Sciences, Cleveland, OH, USA; i Department of Ophthalmology, College of Medicine, The Catholic University of Korea, South Korea; j University of Utah, Department of Ophthalmology and Visual Sciences, Salt Lake City, UT, USA; k Allergan/Abbvie, Ophthalmology Discovery Research, Laguna Niguel, CA, USA; l National Eye Institute, National Institutes of Health, Bethesda, MD, USA; m University of California, Berkeley, Department of Neuroscience, Berkeley, CA, USA; n David Geffen School of Medicine, Stein Eye Institute, Los Angeles, CA, USA; o Duke University Medical Center, Departments of Ophthalmology and of Cell Biology, Durham, NC, USA

**Keywords:** Subgroups of AMD, Diet, Environment, Genetics, Aging, Ancestry, RPE

## Abstract

Clinicians recognize the heterogeneity of age-related macular degeneration (AMD) in presentation, progression, and treatment response, as well as the challenges in distinguishing it from other macular degenerations. As part of the 2024 Ryan Initiative for Macular Research meeting, a group of clinician-scientists and basic scientists were convened to consider the question of whether AMD should be classified as a single disorder or a spectrum of conditions. To answer this question, we reviewed research on several “dimensions” that constitute AMD risk or pathogenesis: genetics, ancestry, retinal imaging findings, diet and environment, aging, and outer retinal molecular and cellular pathways. The group reached a consensus that AMD represents a heterogeneous collection of disease states arising from the interplay of these dimensions. This heterogeneity can be conceived of as a “cloud” of AMD phenotypes. Defining subtypes within this “cloud” requires longitudinal cohorts of well-genotyped and phenotyped patients who progress from no AMD through late AMD, analyzed by unsupervised learning. Comparing the AMD subtypes that emerge from this analysis, especially -omics data from each subtype, will illuminate biology that is applicable to certain subtypes of AMD patients and molecular pathogenic mechanisms that universally apply to all AMD. This knowledge will, in turn, drive improved drug development.

## 1. Introduction

### What is Age-Related Macular Degeneration?

In clinical practice, “macular degeneration” is frequently used to label any progressive outer retinal degenerative phenotype that, if a patient lives long enough, results in geographic atrophy (GA). **Age-related** macular degeneration (AMD), however, is a specific diagnosis and constitutes only a subset of patients with macular degeneration. Historically, criteria for AMD relied on patient age and the presence of particular sizes and shapes of drusen (García-Layana et al., 2017). However, defining AMD, as compared to “macular degeneration” more broadly, is fraught with difficulty. First, drusen can appear in monogenic disorders, especially when age-of-onset is younger. Second, some deposits on clinical imaging and exam can be mistaken for drusen despite being clearly different histologically, including pigment clumps seen in pattern dystrophy and vitelliform deposits seen in a variety of macular degenerative conditions. Third, the pathology of advanced macular degeneration, including AMD, often obscures prior findings, giving rise to convergent appearances that can obfuscate the nature of the instigating disease. Finally, over the past 15–20 years, an entirely different deposit, termed subretinal drusenoid deposit (SDD), whose clinical imaging correlate is called reticular pseudodrusen (RPD), has been increasingly appreciated as important for AMD progression and phenotype (Spaide et al., 2018). Barely visible on standard clinical exam, these deposits between the retinal pigment epithelium (RPE) and neural retina are now frequently seen in AMD during routine multimodal imaging but can be seen in other macular degenerative conditions as well. Thus, no consensus exists on whether AMD can be defined by the exclusive presence of RPD/SDD.

### Why Defining What Constitutes AMD is Important:

In spring 2024, a task-group was assembled at the Ryan Initiative for Macular Research (RIMR) to define whether AMD is one or multiple diseases. This understanding is crucial because AMD subtypes may have distinct causes, underlying biology, and treatment responses. Defining AMD subtypes should improve disease modeling and targeted drug development.

### What Does Constitute AMD:

**Fig. 1.**
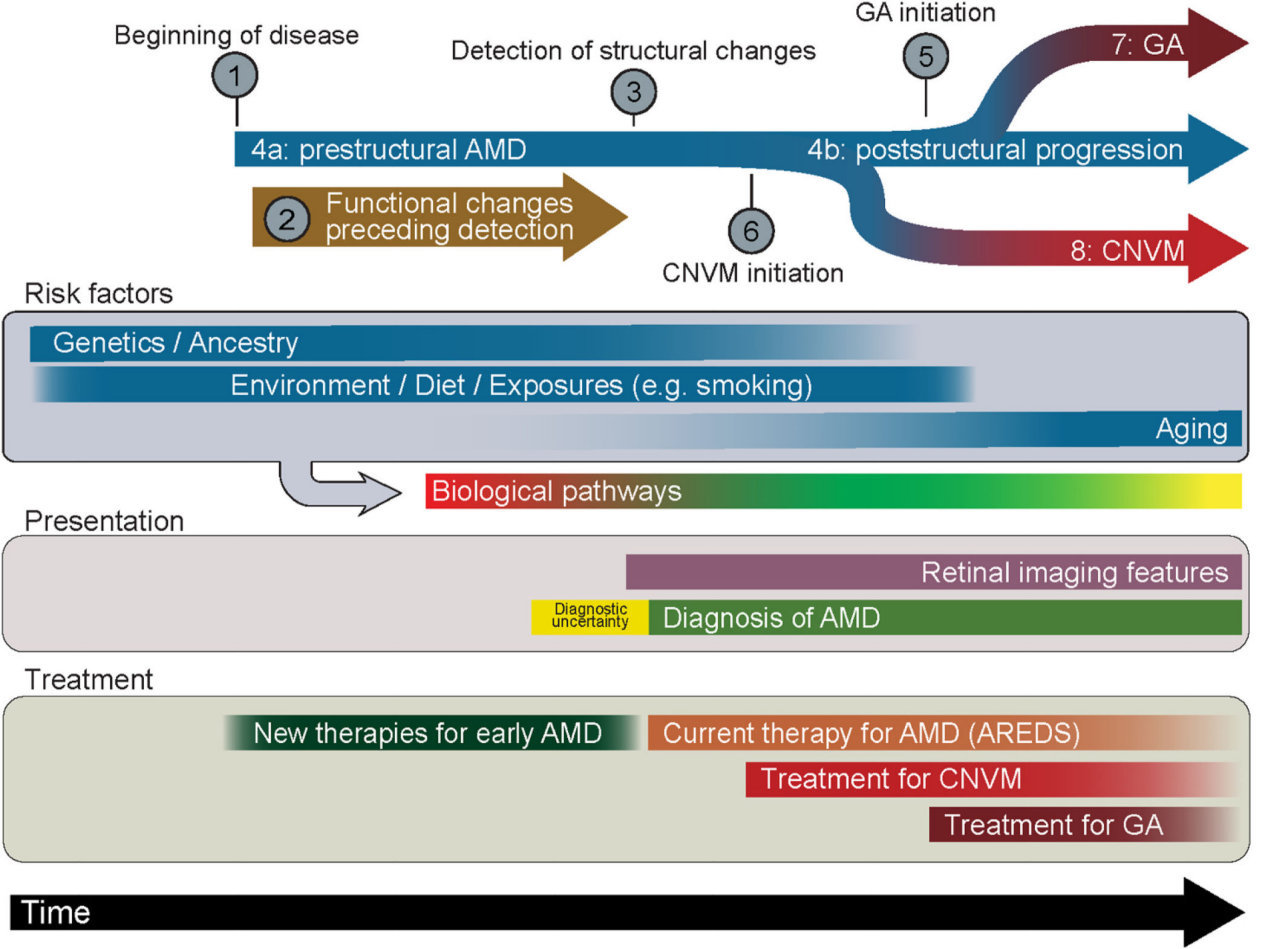
Interrelationships Between AMD Risk Factors, Presentation, Progression, and Treatment AMD can be conceived of having a pre- and post-structural phase. Possible contributions of AMD risk factors to each phase are delineated by colored lines below. Branch-points in AMD progression, including entry of disease into a distinct subtype, are marked in the schematic above. Some branch points, like GA progression or neovascularization, effectively “erase” the prior history leading up to that phase of disease. Whether risk factors are important for predicting only certain phases or subtypes of the disease is unknown. The detailed interrelationships between risk factors is also unknown. Finally, whether retinal features during the post-structural phase carry more explanatory power for predicting ensuing fate than any other AMD risk factors has not been determined.

While the typical definition of AMD involves the buildup of drusen of a certain size in the macula, this simplistic definition fails to consider myriad risk factors, varying disease pathology, and highly dispersed rates of progression (Fig. 1). Decades of research have identified numerous clinical features of AMD, as well as genetic, ancestral, environmental, and cell biological factors that impact the onset, severity and progression of AMD. Yet no single parameter can explain all of the variations that are observed among AMD patients and many AMD-related risk factors are themselves interrelated. For instance, aging and smoking, two risk factors strongly linked to AMD, both cause RPE metabolic and oxidative stress.

### What Is Definitively Not AMD:

Certain macular degenerations should be definitively excluded from being defined as AMD: (1) Monogenetic disorders that lead to drusen accumulation well before age-of-drusen-onset in typical AMD and that are clearly distinct from the overall clinical course of AMD. These include Sorsby’s dystrophy (*TIMP3* mutation), Doyne Honeycomb dystrophy (*EFEMP1* mutation), and Late-Onset Retinal Degeneration (L-ORD, *C1qTNF5* mutation). (2) Pheno-copies for AMD, including central serous choroidopathy, pattern dystrophy, and adult-onset vitelliform dystrophy, can be excluded with careful analysis of multimodal imaging. (3) Patients with drusen exclusively outside the central 6 mm ETDRS grid surrounding the fovea do not have AMD. (4) Late-stage macular degeneration, characterized by neovascularization or GA, cannot be ascribed specifically to AMD unless intermediate AMD characteristics can be identified in the fundus or historical images. (5) The presence of pure RPD/SDD or RPD/SDD with vitelliform material should be acknowledged as ambiguous. Vitelliform material is autofluorescent, extracellular, subretinal material hyperreflective on OCT that represents accumulated shed photoreceptor outer segments secondary to poor RPE uptake. While some patients with RPD/SDD plus vitelliform material may have a Mendelian macular degeneration, others have similar genetic susceptibility and clinical phenotype/fate as “typical” AMD patients (Fragiotta et al., 2024; Naysan et al., 2016, p. 2; Spaide et al., 2019).

### Excluding Late-Stage AMD Progression from Analysis of AMD Subtypes:

**Fig. 2.**
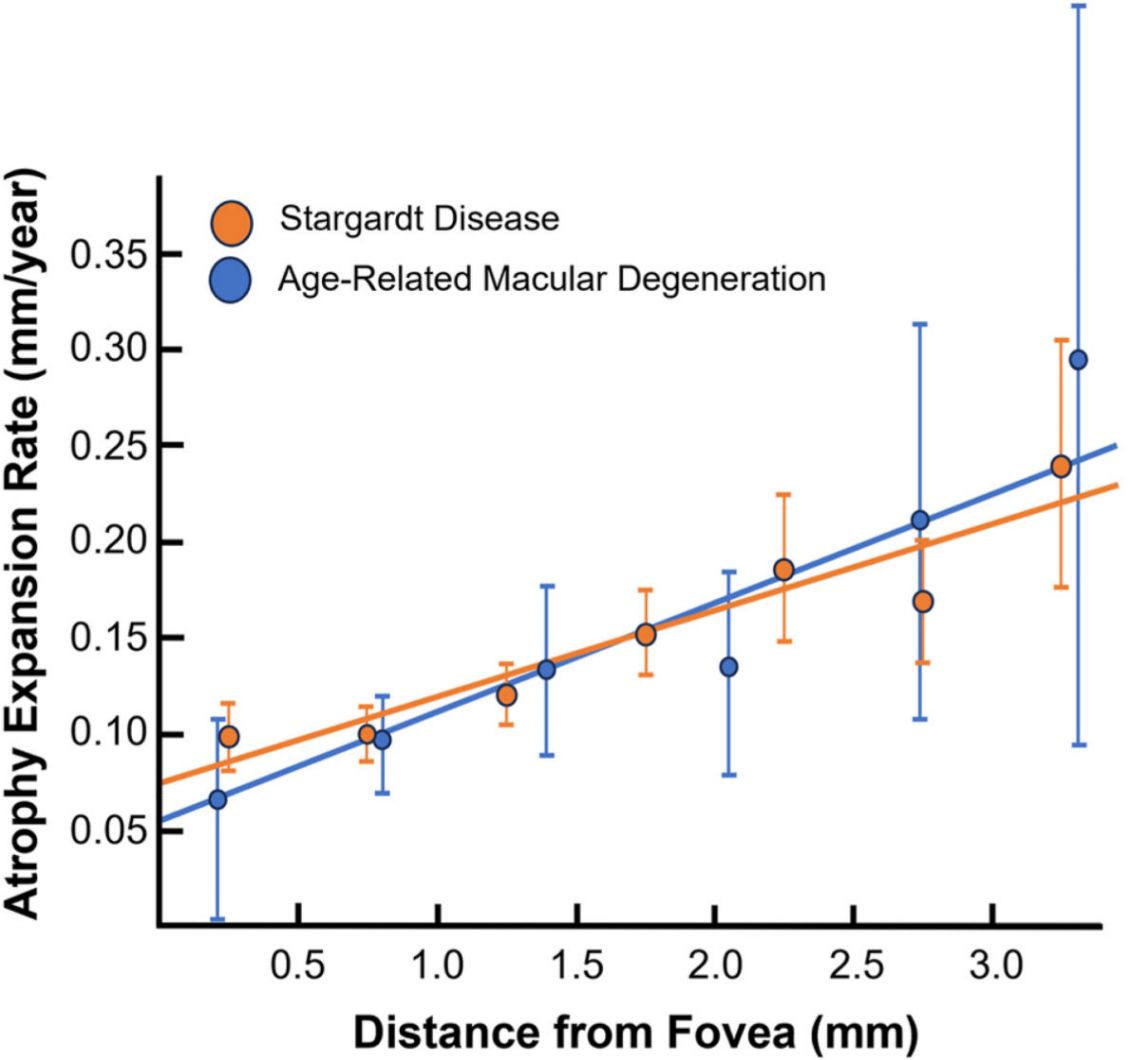
Geographic atrophy (GA) Expansion Rates Are Similar in Different Macular Degenerations. The GA expansion rate for AMD versus Stargardt disease is remarkably similar despite the instigating diseases having significantly different pathogenic mechanisms, with expansion rate highly dependent on distance from the foveal center. This strongly suggests GA expansion is divorced from a patient’s prior history (the entire timeline up until GA onset in Fig. 1) and is a primary reason the Task Group excluded GA expansion from consideration of AMD subtypes. Data extrapolated from the following references (Shen et al., 2021; Young et al., 2023).

For our analysis of AMD subtypes, we excluded the progression of AMD *after* onset of the late-stages of the disease, defined by the start of GA or neovascularization. This decision was made because the fate and tempo of degeneration in late-stage disease is no longer strongly determined by the factors that define AMD in the first place. For example, the type of neovascular membrane rather than the underlying genetics better predicts outcomes once neovascularization starts (Airaldi et al., 2022; Solinsky et al., 2024). In GA, atrophy expansion rates are nearly completely dissociated from AMD genetic risk. Indeed, the largest meta-analysis of genome-wide association studies (GWAS) assessing for GA expansion rates found no association between any single nucleotide polymorphisms and GA progression. None of the 34 key loci previously associated with risk of late AMD *onset* (Fritsche et al., 2016) showed a link to GA *progression*. Furthermore, polygenic AMD risk scores also showed no link to GA progression (Stockwell et al., 2024). Indeed, the progression of GA may represent a self-perpetuating cell-death process that is independent of instigating disease (Csaky et al., 2024). This conclusion is supported by the identical rates of GA progression for AMD versus a distinct monogenetic macular degeneration, Stargardt disease) (Fig. 2).

### Conceiving AMD as Clouds of Phenotypes: Are the clouds well defined or are their edges blurred?

**Fig. 3.**
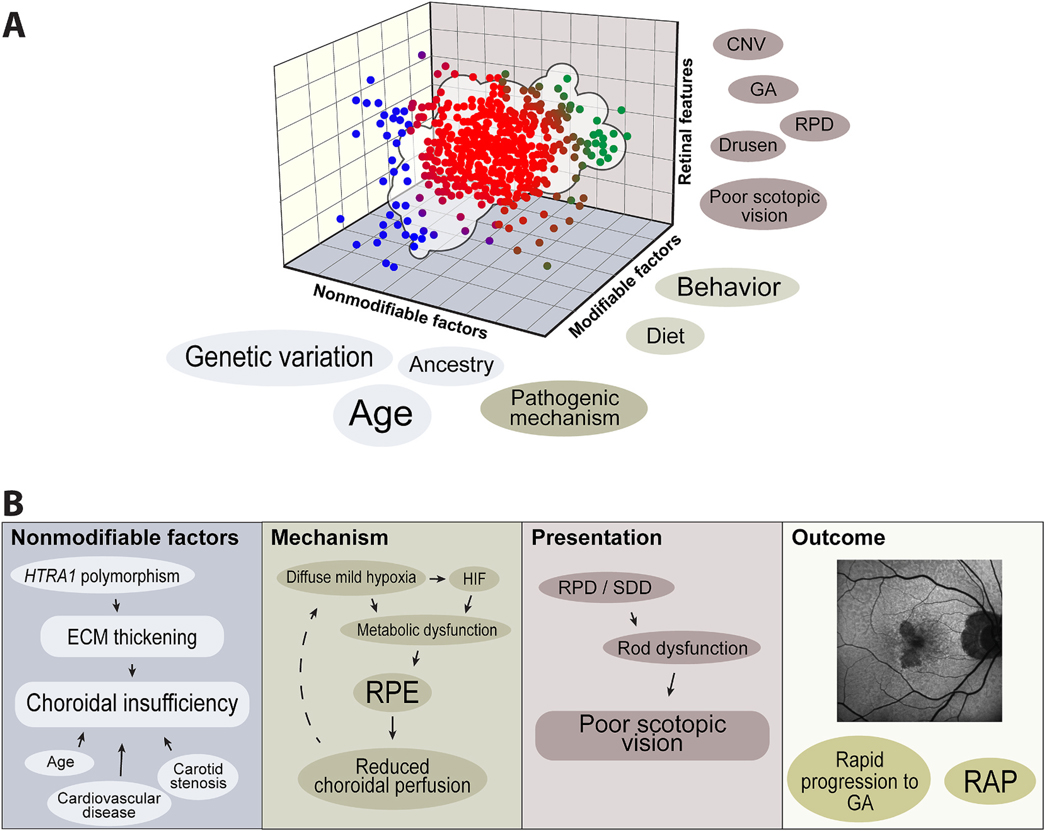
Defining AMD as Clouds of Subtypes in Multidimensional Space. **(A)** Axes that define the multidimensional space where AMD “lives” are risk factors or characteristics well-linked to AMD. These can be broadly broken down into modifiable and non-modifiable risk factors, pathogenic mechanisms, and retinal features (clinical presentation). Axes interact in interdependent ways to create a hyperdimensional space where AMD, AMD mimickers, and monogenic macular degenerations all exist. Points in blue rest at the edge of the AMD cloud and represent likely monogenetic disorders. Points in green suggest the clustering of an AMD subtype in the broad AMD cloud. How these axes combine to define subtypes within the AMD “cloud” is not understood. (**B**) Example of a potential subtype of AMD within the cloud with interdependent axes. Genetic and systemic factors collude to produce either choroidal insufficiency or a thickened extracellular matrix (ECM) that induces mild hypoxia and restricted glucose diffusion across Bruch’s. This, in turn, alters RPE metabolism, either through HIF (hypoxia-inducible factor) or HIF-independent mechanisms. Altered metabolism and mild hypoxia disproportionately affect rods and have also been linked to RPD/SDD. In turn, these features are associated with poor scoptopic vision, rapid progression to GA, and RAP lesions.

The group conceived of AMD as clouds of phenotypes in multidimensional space (Fig. 3A). Each axis of the multidimensional space is defined by key factors linked to AMD (genetics, ancestry, retinal features, environment/diet, aging, and outer retinal biological pathways). Towards the edges of the cloud, the lines between AMD and other macular degenerations blur. Towards the center of the cloud, the AMD phenotype is more certain. From this conceptual framework, the major question the group then asked is whether AMD represents a single cloud or a cluster of clouds (subtypes). By analogy, from a distance, clouds can be seen as distinct entities, but their boundaries become indistinct up close. The goal of this review is to define each axis that makes up the multidimensional space of the AMD cloud(s) and how one can discern whether AMD subtype clouds may emerge from a more comprehensive examination of these axes.

## 2. Characteristics of the axes that define the multidimensional space where AMD phenotypes “live”

The RIMR group divided the risk factors and features that predict AMD onset and progression into six axes, which can be thought of as the coordinates for a hyperdimensional space that spans normal retinal physiology, non-AMD macular degenerations, AMD-mimickers, and true AMD. In a certain portion of this hyperdimensional space rests a cloud of points that defines AMD. Below, we explore how each axis in Fig. 3A may contribute to an AMD subtype, keeping in mind that no single axis can define the entire AMD cloud and that the axes are interdependent.

### 2.1. Genetics Axis

#### Role in Defining AMD Stages and Subtypes:

Genetic analysis has led to the identification of three main pathways implicated in AMD: complement (*CFH-CFHR5, C2/CFB, CFI, C3, TMEM9*7/VTN), lipid homeostasis (*APOE, LIPC, CETP*), and extracellular matrix remodeling (*COL4A3, COL8A1, SYN3/TIMP3, VEGFA ADAMTS9-AS2, ARMS2/HTRA1*) (Colijn et al., 2021; Fritsche et al., 2016) (See also https://www.sciencedirect.com/journal/experimental-eye-research/vol/254/suppl/C Genomics paper in this special issue.). Variants in the *CFH-CFHR5* extended region (Chr1-associated AMD locus) and *ARMS2/HTRA1* region (Chr10-associated AMD locus) (Pappas et al., 2021) account for approximately 70% of the variability in AMD explained by genetic effects (Fritsche et al., 2016), and close to 90% of individuals with AMD carry at least one risk allele at one of these loci (Colijn et al., 2021; Pappas et al., 2021).

Hageman and colleagues employed a “genetic outlier” approach to elucidate the specific, differential contributions of the *CFH-CFHR5* and *ARMS2/HTRA1* loci to AMD phenotype by studying patients and human donor eyes carrying risk at the Chr1 locus only, the Chr10 locus only, or no risk at either locus. They show that Chr1 and Chr10 drive different ocular biologies (Pappas et al., 2021), with Chr10/*HTRA1*-mediated disease linked to faster progression, increased risk of wet AMD (especially type III neovascularization, also called a retinal angiomatous proliferation (RAP), which originates in retina rather than choroid), and the presence of histologic features associated with poor Bruch’s permeability, including basal laminar deposits (BLamD), Bruch’s membrane thickening, and RPD/SDD (Schmitz-Valckenberg et al., 2022; Solinsky et al., 2024; Williams et al., 2021).

At the border of the AMD cloud rests monogenetic disorders, which can help define how a particular gene or process may be involved in “typical” AMD. For example, rare, highly penetrant pathogenic variants in *CFH* cause abundant drusen nearly indistinguishable from typical AMD seen in Chr1-driven disease. This genotype-phenotype relationship is strong enough to dominate over any other AMD modifiers, allowing one to “see” how a pure “CFH risk” determines AMD phenotypes (Kersten et al., 2017).

#### Outstanding Questions Related to Genetics Axis:

Despite notable efforts above, defining each genetic perturbation’s role in specific AMD phenotypes has been difficult. Many studies collapse subgroups of AMD phenotypes into a single phenotype to improve study power at the expense of linking specific genetics to specific AMD subtypes. Further, many genetic associations are derived by comparing an AMD cohort to a control cohort, rather than comparing one AMD phenotype to another AMD phenotype. This approach limits our capacity to link genetics to AMD subtypes. Unfortunately, fine phenotyping of AMD requires huge amounts of labor and time, and very few datasets contain this level of longitudinal detail combined with genetics, which limits our ability to link genotype to AMD subgroups. Lastly, genetics is often considered independently of the other AMD axes below, but these axes may dampen or accentuate the effect of genetics on AMD phenotypes.

### 2.2. Ancestry Axis

#### Role in Defining AMD Stages and Subtypes:

AMD is significantly more common in individuals with European ancestry than those of Asian, African, or Hispanic ancestry (Fleckenstein et al., 2024; Klein et al., 2011; Mahr et al., 2018; Wong et al., 2014). Compared to individuals with European ancestry, drusen in African-Americans tends to be less central (Sadigh et al., 2015). Wet AMD in those of Asian or African ancestry is more likely to manifest as polypoidal choroidal vasculopathy (PCV), a distinct neovascular entity (Fukuyama et al., 2022; Kokame, 2024). Individuals outside of European ancestry also have a lower rate of GA (Rim et al., 2020). Ancestry-segregated GWAS analysis suggests that Chr1/*CFH* and Chr10/*HTRA1* variants impart a different magnitude of AMD risk depending on ancestry. Some of this differential risk may stem from a dependency of *HTRA1* risk on the presence of the *CFH* risk allele, and a lower prevalence of the *CFH* risk allele in the African-American population (Gorman et al., 2022).

#### Outstanding Questions Related to Ancestry Axis:

A more comprehensive analysis of AMD genetic susceptibility loci and differences in environmental factors (diet and smoking, in particular) in those of Asian and African ancestry is needed. Such analysis would help determine if differences in both AMD susceptibility and phenotype in these populations can be attributed to known AMD risk factors, or whether novel behavioral, genetic, or molecular pathways that segregate with ancestry should be probed (for example, ancestral differences in RPE/choroidal melanin or foveal pit morphology (Zouache et al., 2020)).

### 2.3. Retinal Features Axis

#### Role in Defining AMD Stages and Subtypes:

Historically, drusen have been singularly important in defining AMD; however, the size, shape, location, and composition of drusen vary and can affect AMD phenotypes (Khan et al., 2016). For example, while many have drusen and RPE changes in the retinal periphery (Domalpally et al., 2017), these features must be present in the macula to constitute AMD. Calcified drusen, seen with hyporeflective cores on OCT, are more predictive of progression to atrophy (He et al., 2024). Whether calcification simply represents more long-standing drusen (Goh et al., 2022) or a unique calcification program that increases risk for overlying RPE (Rajapakse et al., 2020) is unknown. Other particular features of drusen, including proximity to the fovea, height of the drusen, and presence of hyperreflective foci over the drusen, have all been linked to risk of late AMD (Au et al., 2022; Hirabayashi et al., 2023).

RPD/SDD, found in up to half of AMD patients 85 years and older (Bikbov et al., 2024), have emerged as another important deposit in AMD phenotyping. Localized adjacent to the apical side of the RPE and with a different molecular composition than drusen (Spaide et al., 2018), the presence of RPD/SDD, normalized to equivalent drusen burden, has been linked to all of the following: older age, higher risk of GA onset, earlier deficit in rod-mediated functions, lower choroidal perfusion, and onset of RAP neovascularization (Chen et al., 2019; Digsby et al., 2024; Domalpally et al., 2019; Goerdt et al., 2024; Mordechaev et al., 2024; Sawa et al., 2014). Interestingly, RPD/SDD have also been linked to Chr-10-*HTRA1*-mediated disease (Thee et al., 2022). Thus, the following schema could define a subtype in the AMD “cloud” – *HTRA1* (genetics axis) predisposes to extracellular matrix remodeling that thickens Bruch’s and impairs RPE maintenance of the choroid. This creates a choroidal hypoperfusion state across the whole macula, inducing non-toxic and mild RPE hypoxia (biology axis, see below). The diffuse choroidal hypoperfusion, in turn, triggers formation of RPD/SDD (retinal feature axis) (Digsby et al., 2024), resulting in specific rod dysfunction (functional outcome) and a distinct late AMD phenotype (early GA formation and RAP lesions) (Fig. 3B). It is possible an alternative schema would emerge if connections between axes that define the AMD cloud were instead analyzed by artificial intelligence (AI) in an unsupervised fashion (see Conclusions section). While RPD/SDD have generally been linked to choroidal hypoperfusion, there are reports of SDDs forming above thickened choroids. In such cases, choriocapillaris perfusion may still be limited (a known feature of pachychoroid disorders) or other factors may contribute to RPD/SDD formation (Sacconi et al., 2024).

Another imaging characteristic consistently linked to AMD progression is the presence of longitudinally extensive, proteinaceous basal laminar deposits (BLamD) on the basal side of the RPE (Hirabayashi et al., 2023). Unfortunately, these deposits are better characterized by histology than clinical imaging with current OCT resolution, precluding more detailed association between BLamD and specific AMD subtypes. However, monogenetic diseases involving Bruch’s extracellular matrix remodeling, such as Sorsby’s, Doyne Honeycomb, and L-ORD, all demonstrate thick BLamD, suggesting BLamD signifies poor extracellular matrix remodeling (Sura et al., 2020).

#### Outstanding Questions Related to Retinal Features Axis:

**Fig. 4.**
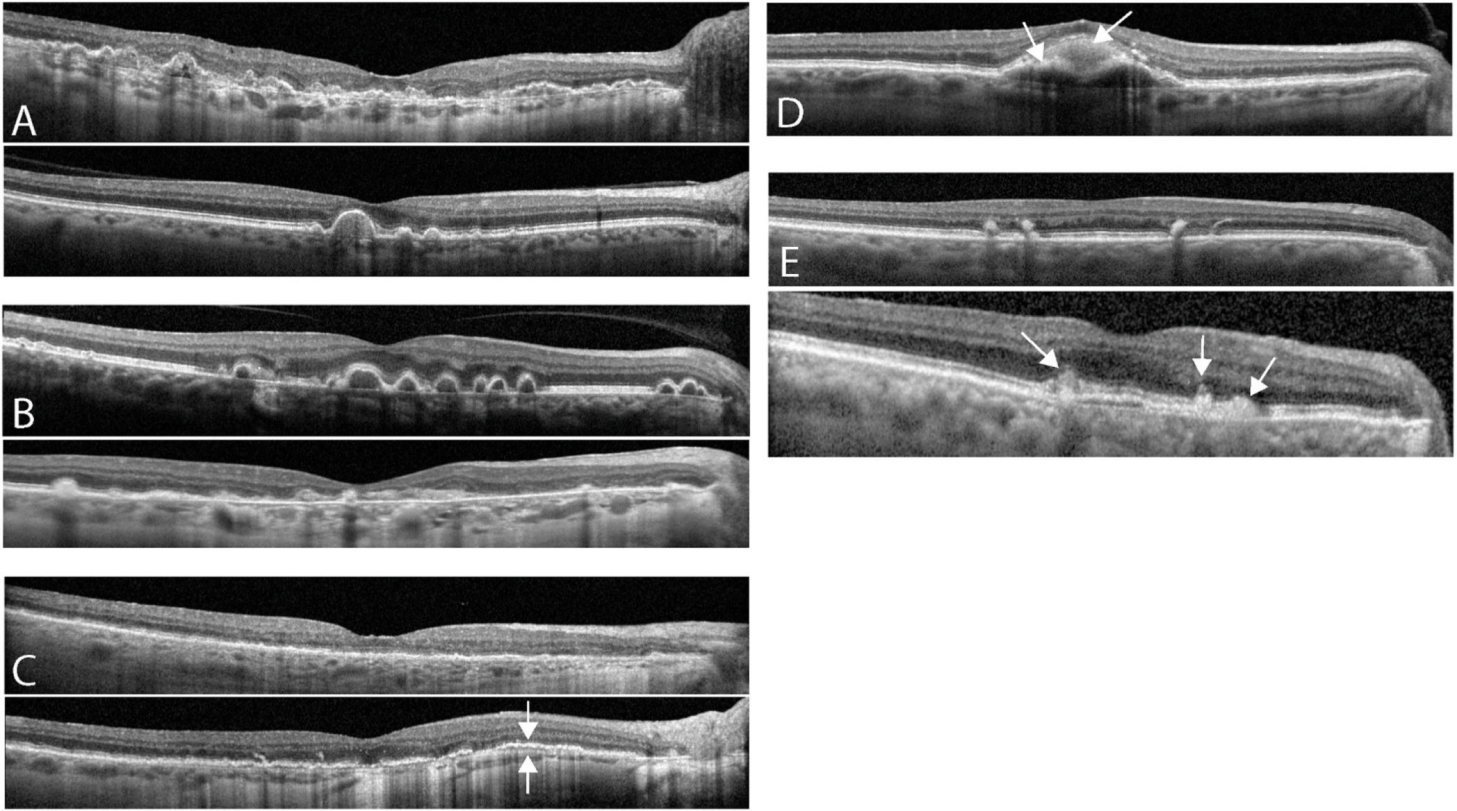
Examples of the Diversity of AMD Retinal Features and AMD Mimickers. (**A**) Drusen can exist throughout the macula (top) or remain solely in the subfoveal and parafoveal regions (bottom) with preserved retinal integrity elsewhere. Assuming similar subfoveal drusen volumes, it is unknown if differences in extent of drusen in the peripheral macula alters AMD subtype or progression. (**B**) Differences in drusen shape and reflectivity. Colloidal-like drusen (top) have a distinctly hyporeflective core that is different from the hyporeflective features of calcified drusen. Other drusen are highly uniform and hyperreflective throughout their core (bottom). These differences in reflectivity likely derive from drusen composition differences, but the link between each drusen type and AMD subtype or progression rate is not well defined. (**C**) Advanced RPD/SDD with minimal drusen burden (top) versus with additional splitting of RPE-Bruch’s complex, indicating presence of BLamD (bottom arrows pointing to Bruch’s and RPE respectively). Whether the additional formation of BLamD in these cases signifies a distinctly different AMD subtype or just a faster progression to GA is unknown. (**D**) Formation of vitelliform material over a drusen. Right arrow points to subretinal vitelliform material while left arrow marks the path of the RPE. Vitelliform frequently forms in AMD and usually signifies worsened prognosis, but its specific link to an AMD subtype or any particular biological pathways, diet or smoking status, or other axes that create the AMD cloud is not well-defined. (**E**) AMD-mimicker: Example of pigment clumping in pattern dystrophy, which can be mistaken for drusen. Pigment clumps (arrows) do not “push up” the RPE, have a more hyperreflective core on OCT imaging, and appear pigmented on clinical exam. Pigment clumping is just one example of many retinal features that can mimic AMD, but which can be readily distinguished from true AMD via careful analysis of multimodal imaging.

How does the myriad of deposit characteristics link to AMD subtypes? For example, some patients only have drusen extending from the fovea to the parafovea while others have drusen extending all the way from the fovea to the perifovea (Fig. 4). Are there differences in fate of these two types of drusen distributions if they have the same volume of foveal drusen? Other patients have different drusen morphologies, likely signifying different compositions, including extreme cases like colloidal (Chen and Chang, 2023) or cuticular drusen (Balaratnasingam et al., 2018) (Fig. 4). Do these different drusen morphologies matter? Other imaging features associated with AMD include vitelliform deposits. The link between other axes in the AMD “cloud” and vitelliform, as well as how well vitelliform defines a unique AMD subtype, remains to be determined (Lindenberg et al., 2024) (Fig. 4).

A final outstanding question is whether the features visible on clinical imaging in intermediate AMD can predict subtypes, defined by particular progression rates, functional deficits, and type of late-AMD, independent of any other axes discussed here. In other words, do all other axes help determine the clinical appearance of AMD in the intermediate stage, but once this stage is reached, the clinical features themselves are then fully capable of predicting or differentiating these disease subtypes? For example, the simple grading of intermediate AMD patients based on deposits and RPE pigment changes can predict progression to late-stage AMD well enough that addition of demographic, environmental, or genetic risk scores add almost no additional predictive power (Ding et al., 2017). If the fundus appearance does not fully predict AMD subtypes once the intermediate AMD stage is reached, is this because other axes are important for prediction or is it because our current clinical imaging is insufficient to detect more refined retina structural features? For example, with emerging high-resolution OCT, fine splitting between Bruch’s and RPE may be more visible than before, allowing more subtle BLamD to be imaged, and thereby offering better resolution on the relationship of BLamD and AMD subtypes (Chen et al., 2023).

### 2.4. Environmental (smoking/diet) axis

#### Role in Defining AMD Stages and Subtypes:

Cigarette smoking is the strongest modifiable risk factor for AMD (Myers et al., 2014; Seddon et al., 1996; Tan et al., 2007; Vingerling et al., 1996), affecting the RPE via multiple mechanisms involving oxidative stress (Handa et al., 2019) and epigenetic modulation (Gao et al., 2016; Joehanes et al., 2016). Smoking increases risk of AMD at all stages of disease, including the transition from no AMD to early AMD (Jonasson et al., 2014; Myers et al., 2014) and from established AMD to either GA or neovascularization (Joachim et al., 2015; Kuan et al., 2021; Smith et al., 2001). Studies are conflicting on how much smoking affects AMD independent of genetic AMD susceptibility. For example, among patients with early AMD and genetic susceptibility, smoking is associated with additive, but not synergistic risk of progression (Baird et al., 2008; Joachim et al., 2018). In contrast, another study suggests genetic variants can strongly impact the relationship between smoking and AMD (Naj et al., 2013).

Diet is another significant modifiable AMD risk factor. While a Western diet increases risk of early and late AMD, a Mediterranean diet is associated with decreased risk (Chiu et al., 2014; de Koning-Backus et al., 2019; Keenan et al., 2020). An interaction between diet and genetics may again be present, as the Mediterranean diet was principally protective in patients without risk at the Chr1-*CFH* locus (Merle et al., 2015). Nutrients may have protective effects that are dependent on stage and location of disease. For example, anti-oxidants and lutein/zeaxanthin specifically protect against expansion of GA to the foveal center, as these micronutrients may fortify the protective effects of the foveal-centered luteal pigments (Keenan et al. 2024).

Outside of a Mediterranean diet, there is strong evidence that low glycemic index (LGI) diets are protective against AMD (Agron et al., ´ 2021; Chiu and Taylor, 2011) while high glycemic index diets are linked with a nearly 3-fold increased risk for early AMD and over a 40% increased risk for late AMD (Bejarano et al., 2024; Chiu et al., 2007).

#### Outstanding Questions Related to Environmental/Diet Axis:

The effects of smoking on RPD/SDD versus drusen, on rod vs. cone function, and other relevant AMD subtypes is not established. A high priority for AMD lifestyle research is to define the AMD subtypes that disproportionately benefit from a Mediterranean or LGI diet.

### 2.5. Aging Axis

#### Role in Defining AMD Stages and Subtypes:

Age is the strongest risk factor for AMD, but it is not entirely clear if AMD represents a distinct disease entity or simply the acceleration of an aging process (See also https://www.sciencedirect.com/journal/experimental-eye-research/vol/254/suppl/C aging article in this special issue of the journal). Aging in the outer retina is marked by diminished choriocapillaris perfusion, Bruch’s thickening (especially with increasing lipids), and rod photoreceptor loss (especially parafoveal rods) but cone preservation (Curcio et al., 2024; Marshall et al., 1979; Sacconi et al., 2019). These features overlap with the pathology of early AMD. While the RPE is lost in AMD, it is preserved but dysmorphic with aging (Curcio et al., 2024; D et al., 2022). The increasing presence of RPD/SDD in very old, otherwise normal eyes (Chan et al., 2016) suggests that aging versus pathological processes governing RPD/SDD may overlap. Early evidence suggests that nutrient deficiency may play a role in RPD/SDD in both aged normal and AMD eyes (Digsby et al., 2024). Furthermore, the association of decreased choroidal perfusion with both normal aging and multiple stages of dry AMD suggests choroidal perfusion state may be another critical link between aging and AMD (Sacconi et al., 2021).

#### Outstanding Questions Related to Aging Axis:

Given the many overlapping features between outer retinal aging and early AMD, a relevant question is whether an individual with a particular accelerated aging of one aspect of the outer retina will manifest with a distinct AMD subtype? To answer this question, we need longitudinal high-resolution imaging and functional assessment of photoreceptors, RPE, and choriocapillaris of aging patients prior to onset of AMD. For instance, a testable hypothesis is that early, severe, choroidal thinning associated with specific rod dysfunction (Prins et al., 2024) will segregate with AMD characterised by marked RPD/SDD formation, whereas other patterns of aging might predict emergence of soft drusen.

The link between age-related changes relevant for AMD and other risk factors for AMD, such as diet, environment, and genes, is largely undefined. Do these other axes that define the AMD “cloud” just set the stage for retarded versus accelerated outer retinal aging, or do they specifically affect AMD susceptibility and progression without affecting normal outer retinal aging? As a corollary, for AMD patients with early age-of-onset of disease, are their risk factors simply accelerating outer retinal aging or are they promoting features of AMD not shared with aging?

### 2.6. Biological Pathways Axis

#### Role in Defining AMD Stages and Subtypes:

The Task Group defined the RPE as a critical cellular “pinch point” for pathogenesis at all AMD stages and phenotypes (Bird, 2021). However, the RPE itself depends on other tissues for support and the relationship between the RPE, photoreceptors, Bruch’s membrane, and the underlying choriocapillaris is critical for the survival and function of all four tissues. Changes in all four tissues are seen in early AMD, including photoreceptor dysfunction, RPE dysmorphia over drusen, thickening of Bruch’s membrane, and reduced choriocapillary area (Curcio et al., 1996; Guymer et al., 1999; Kar et al., 2023; Lutty et al., 1999; Mullins et al., 2011; Pauleikhoff et al., 1999). The parallel pathology observed in all outer retinal layers has made it difficult to determine if RPE represents the “first hit” of AMD, or if the initial insult or originating tissue is the same in all patients. In AMD dominated by drusen versus RPD/SDD, the initial affected cell type may differ. For example, RPE are dysmorphic early in drusen formation specifically over drusen deposits (Gambril et al., 2019; Hammer et al., 2021), progressing to a phenotype of epithelial-to-mesenchymal transition (EMT) involving migration off the RPE monolayer, reflected as “high-risk” hyperreflective foci (HRF) on OCT imaging, which immediately precedes onset of total RPE loss (GA) under the drusen with HRF (Zhang and Miller, 2022). In contrast, the formation of RPD/SDD leads preferentially to photoreceptor death, as demonstrated by outer nuclear layer thinning on OCT, prior to frank onset of RPE death, as marked by GA onset (Chen et al., 2020; Spaide, 2013).

Biological pathways postulated to play a role in AMD are numerous: oxidative stress and antioxidant systems, phagolysosomal and autophagic systems, lipid handling and toxicity, glycolysis and mitochondrial metabolism, innate immunity, hypoxia, extracellular matrix remodeling, cell death pathways, and EMT, among others. Almost all these pathways are interrelated, with oxidative stress, for example, having established effects on autophagy, metabolism, innate immunity, cell death, and EMT.

#### Outstanding Questions Related to Biological Pathways Axis:

A particularly important question is whether one or more cell types or biological pathways dominates in all AMD disease or a particular stage or subtype of AMD. This would strongly influence whether a clinical trial testing a specific biological mechanism of action should be applied to all patients with AMD or a select AMD stage or subtype. Certain cell types or biological pathways may be specifically critical for a particular AMD subtype. For example, in Fig. 3B, choroidal insufficiency is linked to RPD/SDD formation and rod dysfunction. Rod function tightly correlates with choriocapillaris perfusion (Prins et al., 2024). This correlation suggests the following biological mechanism may come into play when rod function is particularly affected early in AMD or when disease is dominated by RPD/SDD: subtle hypoxia stimulates the hypoxia inducible factor (HIF), a transcription factor that alters RPE metabolism towards a glycolytic state. This forces the RPE to consume glucose, but glucose availability may also be low due to poor diffusion through a thickened Bruch’s complex seen in aging. The lower glucose availability, combined with HIF-driven glycolysis in the RPE, deprives photoreceptors of their preferred metabolic substrate. Rods have been established as more susceptible to glucose deprivation than cones, so HIF activation leads preferentially to rod deficits (Daniele et al., 2022; Kar et al., 2023). Thus, AMD dominated by rod dysfunction or RPD/SDD may specifically benefit from interventions related to blocking activity of HIF or its effectors (Kurihara et al., 2016).

Besides the obvious connection of CFH to innate immunity, HTRA1 to extracellular matrix remodeling, or cigarette smoking to oxidative stress, it is also unknown how other axes that contribute to the AMD “cloud” intersect with biological pathways implicated in AMD. If a particular combination of certain axes consistently points towards a biological pathway being heavily involved in their pathogenesis, then patients with that specific combination may best be treated by targeting the identified pathway.

## 3. Conclusions and recommendations

The RIMR Task Group assembled to answer whether AMD was a single or multiple diseases and concluded that AMD is a spectrum of conditions on a multi-dimensional scale. Factors such as genetics, ancestry, retinal features, environment/diet, aging, and biological pathways relevant to outer retina health all intersect to define AMD’s complexity. Imagining these factors as axes on a hyperdimensional coordinate system, AMD occupies a certain space (or cloud) in the coordinate system. Adjacent to this AMD cloud are monogenetic disorders that closely resemble AMD pathology but whose pathophysiology is dominated by genetic risk. Within the sporadic AMD “cloud(s)”, it is likely that subtypes of AMD exist (Fig. 3A). In this review, we defined some examples of potential subtypes. For example, in Fig. 3B, we defined a cloud subtype that involves *HTRA1*, choroidal insufficiency, hypoxia and glucose deprivation, RPD/SDD, rod dysfunction, type III neovascularization, and early GA. In the aging and biological pathways section, we further elaborated on this subtype by demonstrating it may arise specifically in the oldest AMD individuals and involve HIF transcription factor signaling.

Despite the seeming blurring of one AMD subtype into another, there is remarkable symmetry in the onset, lesion localization, and progression characteristics between the two eyes of a patient (Mann et al., 2011). This implies that specific phenotypic characteristics for a patient (their location in the AMD “cloud”) is a predictable consequence of the patient’s risk factors rather than a stochastic process. However, our ability to delineate subtypes in the AMD “cloud(s)” is currently limited by incomplete data and the inability of our minds to integrate all the interrelationships between the axes. To genuinely define AMD cloud subtypes, we need to apply artificial intelligence on large datasets in which data from each of the axes above is recorded.

How do we go about this task? Ideally, a collection of older subjects is followed longitudinally over time in which each person is comprehensively characterized: smoking status, diet questionnaires, genetic testing and other omics datasets, epidemiologic information, and continuous structural and functional testing sensitive to the earliest changes of outer retina pathology. Rates of AMD progression would be recorded by longitudinally following the cohort.

Using AI on such comprehensive datasets would.

- Allow unbiased AMD subtype identification: Natural subtypes within the AMD “cloud” would be discovered without preconceived notions.
- Enable a connection between biological pathways and AMD subtypes: Comparing “omics” data between different AI-identified AMD subtypes will highlight specific biological pathways important for that AMD subtype.
- Lead to precision therapy: The link between biological pathways and AMD subtypes would then enable the targeting of precise therapies to specific AMD subtypes.

A major question that will be answered with such comprehensive analysis is whether all AMD subtypes and progression rates at the intermediate stage of the disease can be defined simply by retinal imaging. While retinal imaging in intermediate AMD is highly predictive of progression rates and functional deficits as disease advances to late AMD, it is unknown whether a combination of other axes would further augment disease prognostication.

## Data Availability

Data will be made available on request.
